# The Endoscopic Ultrasonography and Fine Needle Aspiration Agreement in Patients with Pancreatic Cystic Lesions, in Tehran, Iran

**DOI:** 10.34172/mejdd.2025.425

**Published:** 2025-04-30

**Authors:** Arman Rahimi Mohseni, Elham Sobhrakhshankhah, Farhad Zamani, Mahmoodrza Khoonsari, Amirhossein Faraji, Mehdi Nikkhah, Ali Ajdarkosh, Fahimeh Safarnezhad Tameshkel, Mehran Haghighi, Gholam Reza Hemmasi, Hossein Ajdarkosh

**Affiliations:** ^1^Gastrointestinal and Liver Diseases Research Center, Iran University of Medical Sciences, Tehran, Iran

**Keywords:** Cystic pancreatic lesions, Endoscopic ultrasound, Cyst fluid analysis

## Abstract

**Background:**

IThis study aimed to investigate the concordance of endoscopic ultrasonography (EUS) results and biochemical analysis of cyst fluid obtained through fine needle aspiration (FNA) in diagnosing mucinous pancreatic cysts.

**Methods:**

In this retrospective study, the medical records of 81 patients with pancreatic cystic lesions were examined. EUS results and cyst fluid analysis (carcinoembryonic antigen [CEA] and amylase) concordance rate was evaluated, and the agreement between the two methods was assessed through Cohen’s kappa coefficient.

**Results:**

The concordance rate of EUS and cyst fluid analysis with CEA cutoff point>192 ng/mL was 52.5% and 69.2%, respectively, for mucinous cystic neoplasm (MCN) and Intraductal papillary mucinous neoplasm (IPMN) diagnosis. The sensitivity and specificity of cyst fluid analysis for MCN and IPMN (taking EUS as the reference) were 59.1% and 93.33%, respectively. The positive predictive value was equal to 97.5%, and its negative predictive value was 34.14. Using the CEA<5 ng/mL cutoff point for detecting serous cystic neoplasm (SCN) cysts was associated with the sensitivity, specificity, and positive and negative predictive values of 73.3%, 78.8%, 44%, and 92.8%, respectively. The concordance rate of EUS and pancreatic cyst fluid analysis was 65.43%, with a kappa correlation coefficient of 0.326.

**Conclusion:**

The specificity and positive predictive value of CEA>192 for the diagnosis of mucinous pancreatic cysts is high, but it is associated with moderate sensitivity and a low negative predictive value. Altogether, there is a moderate agreement between the results of EUS and biochemical analysis of FNA cyst fluid in diagnosing mucinous pancreatic cysts.

## Introduction

 Due to the widespread use of abdominal imaging methods and the aging of the population, the prevalence of pancreatic cystic lesions has increased significantly in recent years. The prevalence of pancreatic cysts larger than 1 cm in the general population is 2%, and their frequency increases with age, reaching 10% in those over 70 years.^1,2^ Pancreatic cystic lesions include a wide range of diagnoses that can be classified into neoplastic and non-neoplastic types. These lesions are highly variable in terms of clinical behavior and malignant potency.^3^ In the past, pseudocysts were considered the cause of 80%-90% of pancreatic cystic lesions, but currently, cystic neoplasms are reported to cause about 60% of these lesions.^4^

 Clinically, it is very important to distinguish neoplastic from non-neoplastic cysts.^5^ Among the imaging methods, endoscopic ultrasonography (EUS) provides high-resolution imaging of pancreatic lesions and is widely used today in evaluating solid pancreatic lesions, non-solid biopsy, and evaluation of cystic pancreatic lesions.^6,7^ When the histological diagnosis obtained from surgery is considered a reference, the diagnostic accuracy of EUS imaging ranges from 40% to 96%, which is highly variable.^8^ Adding fine needle aspiration (FNA) to this method can increase diagnostic accuracy. Ultrasonography-guided fine needle aspiration (EUS-FNA) provides samples for cyst fluid analysis and cytology examination. Although EUS-FNA cytology examination detects tumors with a specificity close to 93%, it has a low sensitivity, which was reported as 54% in a meta-analysis.^9^ The low sensitivity of this method can be due to factors such as obtaining few lesional cells from the aspirate, insufficient sample volume, and sample contamination with gastrointestinal wall cells.^10^ Another option available during EUS-FNA is to analyze markers inside the aspirated cyst fluid. Carcinoembryonic antigen (CEA) and amylase level are the most commonly investigated markers among the various markers.^11^ However, the effectiveness of biochemical analysis of pancreatic cyst fluid obtained through FNA is still debated.^11-13^ Some guidelines support EUS-FNA role,^14^ but others have limited its indications.^15^ A search in reliable domestic and foreign databases showed that, so far, no study has been conducted in the country regarding the concordance of EUS results and biochemical analysis of fluid obtained from FNA in patients with pancreatic cysts. Therefore, the present study was conducted to verify the agreement between EUS results and biochemical analysis of cyst fluid obtained from FNA in diagnosing mucinous pancreatic cysts.

## Materials and Methods

 The current study is a retrospective cross-sectional type. In this study, first, by searching the database of Firouzgar hospital in Tehran, patients who were diagnosed with pancreatic cysts and underwent EUS and biochemical (CEA and amylase) cyst fluid analysis obtained through FNA during 2019-2021 were included. Patients who were diagnosed with pseudocysts in EUS or whose records were incomplete were excluded from the study. Then, the information related to the type of cyst, age, and sex of the patients, as well as the results of the biochemical analysis of the fluid obtained from FNA, were collected from the patients’ records. The samples were selected using available sampling methods.

###  Biochemical Analysis 

 The cyst fluid obtained through FNA was tested to determine its amylase and CEA levels using an enzymatic assay (Pars Azmoon kits, Iran). Based on the suggested cutoff points for CEA and amylase pancreatic cyst fluid to differentiate different pancreatic cysts,^7^ cytological diagnosis was made as follows: (1) Diagnosis of mucinous cystic neoplasm (MCN), as CEA > 192 ng/mL, (2) Diagnosis of intraductal papillary mucinous neoplasm (IPMN) considered as CEA > 192 ng/mL, and by simultaneously considering CEA > 192 ng/mL and amylase > 250 IU/L, 3) Diagnosis of serous cystic neoplasm (SCN) was evaluated by CEA < 5 ng/mL and by CEA < 192 ng/mL.

###  Statistical Methods

 Qualitative descriptive information was expressed as frequency (percentage), and quantitative information was expressed as mean ± standard deviation. Mann-Whitney and chi-square statistical tests were used to analyze quantitative and qualitative data, respectively. Sensitivity, specificity, positive and negative predictive values of different CEA, and amylase cutoff levels of the cyst fluid to diagnose the kind of cyst were calculated considering diagnostic EUS as the reference approach. The concordance rate of EUS and cyst fluid analysis was evaluated, and the agreement between the results of the two methods was assessed using Cohen’s kappa coefficient. The data was statistically analyzed using SPSS software version 21.

## Results

###  Background Findings

 Eighty-one patients (24.7% male) with an average age of 57.09 ± 15.01 years and in the age range of 21 to 98 years were included in the study. According to the EUS report, 40 patients (49.4%) were diagnosed with MCN, 26 patients (32.1%) were diagnosed with IPMN, and 15 patients (18.5%) were diagnosed with SCN. Information related to the age and sex of patients in each type of pancreatic cyst based on the EUS report is shown in Table 1. The three subgroups of diagnosed neoplasms were more prevalent in women, but there was no statistically significant difference in terms of sex distribution among them. Regarding the age of patients, the average age of patients with IPMN was significantly higher than patients with MCN and SCN (*P* = 0.043).

**Table 1:** Background information of included patients based on EUS results

**Variable**		**Cyst type based on EUS results**			***P*** ** value**
		**MCN**	**IPMN**	**SCN**	
Age		54.32 ± 16.72	63.11 ± 11.69	54.06 ± 12.87	0.043
Sex	Male	6 (15%)	10 (38.5%)	4 (26.7%)	0.095
	Female	34 (85%)	16 (61.5%)	11 (73.3%)	

Abbreviations: MCN, mucinous cystic neoplasms; IPMN, intraductal papillary mucinous neoplasms; SCN, serous cystic neoplasms. Mann-Whitney and Chi-square statistical tests were used to analyze quantitative and qualitative data.

###  Biochemical Analysis of Cyst Fluid

 To divide pancreatic cysts into mucinous and non-mucinous cysts, a cutoff point of 192 ng/mL was used for cyst fluid CEA level. 40 individuals (49.4%) had a CEA level of > 192 ng/mL, of whom 39 (97.5%) were diagnosed as having MCN and IPMN, and one (2.5%) was diagnosed as having SCN based on EUS results. Also, 41(50.6%) individuals had CEA level < 192 ng/mL, of whom 14 (34.14%) had SCN, and 27 (65.85%) had MCN or IPMN based on EUS results. So, the positive predictive value of cyst fluid analysis based on the CEA cutoff point > 192 ng/mL for MCN and IPMN was equal to 97.5%, and its negative predictive value was 34.14% (Table 2).

**Table 2:** Statistical indicators of biochemical cyst fluid analysis in differentiating mucinous cysts from non-mucinous cysts regarding EUS as the reference

**Cut Points**	**Sensitivity (%)**	**Specificity (%)**	**Positive predictive values (%)**	**Negative predictive values (%)**
CEA > 192 ng/mL (for MCN and IPMN)	59.1	93.3	97.5	34.1
CEA > 192 ng/mL and Amylase > 250 IU/L (for IPMN)	65.4	45.1	53.8	82.1
CEA < 5 ng/mL (for SCN)	73.3	78.8	44	92.8

MCN, mucinous cystic neoplasms; IPMN, intraductal papillary mucinous neoplasms; SCN, serous cystic neoplasms; CEA, carcinoembryonic antigen; EUS, endoscopic ultrasonography.

 In other words, out of a total of 66 cysts that were diagnosed as MCN or IPMN in EUS, 39 (59.09%) cysts had a CEA level of > 192 ng/mL. Also, among 15 cysts that were diagnosed as SCN in EUS, CEA level was < 192 ng/mL in 14 (93.33%) cysts. Based on this, the sensitivity and specificity of this cut point in the diagnosis of pancreatic mucinous cysts (taking EUS as the reference) were calculated as 59.1% and 93.33%, respectively (Table 2).

 When taking into account both the amylase > 250 IU/L and CEA > 192 ng/mL cutoff points at the same time to diagnose IPMN cysts, 17 out of 26 patients (65.4%) diagnosed with IPMN in EUS also had the same diagnosis in cyst fluid analysis. Sensitivity, specificity, positive and negative predictive values of simultaneous amylase > 250 IU/L and CEA > 192 ng/mL cutoff points to diagnose IPMN (taking EUS as the reference) were 65.4%, 45.1%, 53.8%, and 82.1%, respectively (Table 2).

 Using the CEA < 5 ng/mL cutoff point for detecting SCN cysts, 11 out of 15 patients (73.3%) identified with SCN in EUS had a CEA level < 5 ng/mL, resulting in sensitivity, specificity, and positive and negative predictive values of 73.3%, 78.8%, 44%, and 92.8%, respectively (Table 2).

 The concordance rate between EUS results and cyst fluid analysis based on different cut points is shown in Table 3. Considering CEA > 192 ng/mL cutoff point, the concordance rate was equal to 52.5% and 69.2% for pancreatic MCN and IPMN cysts, respectively. Considering CEA > 192 ng/mL and amylase > 250 IU/L simultaneously, the concordance rate was equal to 65.4% for IPMN cysts. In the case of SCN cysts, the concordance rate, considering CEA < 192 ng/mL and CEA < 5 ng/mL, was equal to 93.3% and 73.3%, respectively. According to these results, the concordance between the two methods was 65.43% in all the examined patients, which shows the Kappa correlation coefficient = 0.326 and indicates an average agreement between the two methods (Table 3).

**Table 3:** Concordance rate between EUS results and biochemical cyst fluid analysis with different cutoff points of CEA and amylase level

**EUS diagnosis**	**CEA>192 ng/mL**	**CEA<192 ng/mL**	**CEA>192 ng/mL and Amylase>250 IU/L**	**CEA<5 ng/mL**
MCN	21 (52.5%)	19 (47.5%)	-	-
IPMN	18 (69.2%)	8 (30.8%)	17 (65.4%)	-
SCN	1 (6.7%)	14 (93.3%)	-	11 (73.3%)
Total	65.43%			

MCN, mucinous cystic neoplasms; IPMN, intraductal papillary mucinous neoplasms; SCN, serous cystic neoplasms; CEA, carcinoembryonic antigen; EUS, endoscopic ultrasonography.

## Discussion

 According to the results of the present study, 75.3% of patients with pancreatic cysts were women. 88% of MCN cysts, 62.5% of IPMN cysts, and 70% of SCN cysts were observed in women, respectively. Although all three types of cysts were more common in women, the sex distribution was not statistically significant for cyst differentiation. Epidemiological studies have also shown that pancreatic cystic lesions are more common in women, so cystic mucinous neoplasms of the pancreas are almost always seen in middle-aged women, and only pseudocysts are more common in men.^16,17^ The higher frequency of these lesions in women is probably due to the role of hormonal factors and estrogen receptors in these lesions.^18^ The mean age of patients with IPMN cysts was significantly higher than patients with MCN and SCN. This result has also been observed in other studies.^16,17^

 The present study’s results show a moderate concordance between EUS results and pancreatic cyst fluid analysis obtained from FNA. About 41% of cases diagnosed as MCN and IPMN by EUS in our study had a cyst fluid CEA level of less than 192 ng/mL (the recommended cutoff point of the American Gastroenterological Association), which indicates the moderate specificity of this method for differentiating mucinous cysts from non-mucinous cysts. However, in contrast, only one sample of non-mucinous neoplasms had a CEA level higher than 192 ng/mL. So, the sensitivity of this marker for differentiating two groups of mucinous and non-mucinous neoplasms was 59.9%, which indicates an average sensitivity for this cutoff point (192 ng/mL for cyst fluid CEA). In the study by Khoury and colleagues, 42.4% of patients with pancreatic mucinous cysts had a CEA level higher than 192 ng/mL, which is close to our results.^16^ In the study of Kurita et al in Japan, the sensitivity of cyst fluid CEA measurement for diagnosing mucinous cysts was 60.9%, and its diagnostic accuracy was 71.8%,^13^ a sensitivity close to our study. A multicenter study in the United States with 120 patients showed that cyst fluid CEA level higher than 192 ng/mL had a diagnostic sensitivity of 75%, specificity of 84%, and diagnostic accuracy of 79% in differentiating mucinous cysts from non-mucinous cysts,^4^ which compared with the present study, reported higher sensitivity and lower specificity. Several other studies have been published about the optimal level of CEA in cyst fluid to identify mucinous cysts, which is in the range of 30 to 480 ng/mL.^19-21^ An older study using a cutoff point of 400 ng/mL for CEA reported a sensitivity of 57% and a specificity of 100% in differentiating between lesions.^22^

 In the present study, for the diagnosis of SCN, the sensitivity of cyst fluid CEA levels < 5 ng/mL and < 192 ng/mL was 73.3% and 93.3%, respectively. Recent studies showed that CEA < 192 ng/mL is associated with about 50% sensitivity and 95% specificity for diagnosing non-mucinous pancreatic cysts, indicating a lower sensitivity compared with our study.^23^ Of course, in the present study, we did not examine pancreatic pseudocyst lesions, which could influence these results. However, more data are needed to determine the optimal cutoff point for CEA in differentiating mucinous from non-mucinous pancreas lesions.

 According to the findings of our study, in 80.8% of individuals diagnosed as having IPMN in EUS, cyst fluid amylase level was > 250 IU/L, and if CEA > 192 ng/mL and amylase > 250 IU/L were considered at the same time, this amount was reduced to 65%. The amylase level of the cyst fluid is an indicator of the connection with the pancreatic duct, so it is mainly used to differentiate the pseudocyst from other types of pancreatic cysts. Failure to investigate pancreatic pseudocysts in our current study can affect the results. But in line with our results, a systematic review analyzing the results of 12 studies (including 450 patients) reported that an amylase level of less than 250 IU/L is associated with a specificity of more than 98% for rejecting the diagnosis of pancreatic pseudocyst.^24^ In another study, an amylase level higher than 479 IU/L was reported with 73% sensitivity and 90% specificity for differentiating pseudocysts from other types of pancreatic cysts.^25^ In summary, further studies are needed to determine an optimal cutoff point for amylase to differentiate pseudocysts from other pancreatic cysts.

 Based on the results of our investigation, the concordance rate between the two approaches was determined to be 73.3% when considering the cutoff point of 5 ng/mL CEA for the diagnosis of SCN. For every participant, the concordance of results between cyst fluid analysis and EUS was 65.4%, which shows the kappa correlation coefficient = 0.326 and indicates an average agreement between the pancreatic cyst fluid analysis and the EUS results. Khoury et al also reported a moderate agreement between EUS results and pancreatic cyst fluid analysis. In this study, there was a poor agreement between the diagnosis of mucinous cysts in EUS with a cyst fluid CEA level higher than 192 ng/mL and a moderate agreement between the diagnosis of SCN with a CEA level less than 5 ng/mL.^16^ So, a more accurate algorithm is needed to reduce various errors and improve the sensitivity in identifying malignant cysts.^26^

 This study is the only study in Iran that compares the results of the biochemical analysis of pancreatic cyst fluid (based on common markers) with the results of EUS. The study’s findings provide valuable insights into the concordance rate of these two methods that can be considered in clinical use. This study also has some limitations. For example, in the present study, we could not use pathology-based diagnosis, the gold standard, because it requires surgery, which was not necessary for many patients with serous or mucinous cysts due to the lack of surgical indications. Instead, we compared endosonographic diagnosis with cyst fluid analysis, a common but less sensitive diagnostic method used in similar studies. Consequently, we could not accurately determine the sensitivity and specificity of endosonography or cyst fluid analysis. Another limitation of the present study is its retrospective and single-center nature. Considering that EUS is a process dependent on the skill of the person performing it, conducting a multicenter study can increase the accuracy of the resulting conclusions.

## Conclusion

 The specificity and positive predictive value of CEA > 192 ng/mL for the diagnosis of mucinous pancreatic cysts is high but it is associated with a moderate sensitivity and a low negative predictive value. Adding amylase > 250 IU/L to CEA > 192 ng/mL, increases the sensitivity and negative predictive value but causes a sharp decrease in specificity and positive predictive value. In general, there is a moderate correlation between endoscopic ultrasound results and biochemical analysis of cyst fluid in the diagnosis of mucinous pancreatic cysts.
